# A mixed methods cross-sectional study of peer support needs and barriers among multiple sclerosis caregivers in Ireland

**DOI:** 10.1177/13591053251405034

**Published:** 2026-01-16

**Authors:** Joan Alaboson, Laura Coffey, Rebecca Maguire

**Affiliations:** 1 Maynooth University, Kildare, Ireland

**Keywords:** informal caregivers, multiple sclerosis, wellbeing, peer support, mixed-methods, barriers, convergent design

## Abstract

Peer support may help improve the wellbeing of informal caregivers, although little work has explored this in the context of multiple sclerosis (MS). This study explored peer support needs and experiences among MS caregivers in Ireland. A cross-sectional mixed methods survey of 27 MS caregivers, designed with public and patient involvement, measured peer support engagement, sociodemographic and care characteristics, caregiver burden, social support, loneliness and wellbeing. Data were analysed using descriptive, correlational and content analysis. Most caregivers expressed a desire for peer support, with few having participated in online or in-person peer support. Barriers to engagement included a lack of promotion of opportunities. While low levels of social support and wellbeing were reported, no clear associations between peer support needs and these variables were identified. Although peer support shows promise, more research and improvements in the provision of MS caregiver peer support are needed.

## Introduction

Living with multiple sclerosis (MS) may present a future of uncertainty and change not only for people with MS (PwMS), but also for their family, friends or close contacts, who often provide non-remunerated support or care to PwMS as informal caregivers (Maguire and Maguire, 2020). Current estimates suggest that up to 46% of family members of PwMS provide informal care across Europe (Kobelt et al., 2017). Caregivers may be involved in assisting PwMS with activities of daily living, potentially supporting physical needs, providing emotional support, coordinating external responsibilities (Borreani et al., 2014), or taking up family roles and responsibilities previously held by PwMS (Meca-Lallana et al., 2016).

The caregiving literature highlights unique caregiver needs related to their roles. For example, caregivers can experience stress, burnout (Holland et al., 2011), and caregiving burden (Meca-Lallana et al., 2016). In caring for PwMS, MS caregivers may be faced with managing their own needs, including the need for psychosocial support (Kinyanjui et al., 2018), as well as those of their care recipients, increasing their risk of experiencing poor wellbeing (Finlayson and Cho, 2008). MS caregivers may go on to face negative consequences from the care they provide including, but not limited to, lower psychological wellbeing (Buchanan et al., 2010; Topcu et al., 2016), depression, lower quality of life (Rajachandrakumar and Finlayson, 2022), social isolation (Borreani et al., 2014) and physical illness, as well as work and financial challenges (McKenzie et al., 2015).

There is a need to explore the potential of interventions to support MS caregivers and mitigate the multidimensional challenges they may encounter. One way in which this may be achieved is through the provision of peer support. Peer support involves an exchange of support between people who have a similar life experience (in this case, support from other MS caregivers; Gerritzen et al., 2022). Peers may connect face-to-face in person, or online through digital internet-mediated communication options, which have become more popular since the COVID-19 pandemic (Boyt et al., 2022).

By engaging with other peers, MS caregivers may experience improved wellbeing. For example, peers may help to provide informational and emotional support, or practical or instructional advice to each other. This has been demonstrated in the context of caring for people with cancer (Dunn et al., 2003) as well as MS (Aterman et al., 2023). The benefits of peer support interventions have been highlighted in the literature. For example, in the context of cancer caregiving, peers can help model coping and other strategies to deal with the multidimensional effects of caring for a person with a long-term illness (Andersen et al., 2019). In neurodegenerative illnesses, caregiver peer support has improved psychological wellbeing, in both face-to-face and online contexts (Marziali and Donahue, 2006). Further, findings of a systematic review and meta-analysis of studies exploring the effectiveness of online psychological interventions, including peer support, suggest improvements in burden, quality of life, and overall wellbeing among caregivers of individuals with neuro-degenerative illnesses (Boyt et al., 2022). Evidence from a scoping review of peer support for informal caregivers across different chronic health conditions suggests its utility in bridging gaps unmet by healthcare teams relating to the provision of relevant skills, information, and emotional supports to caregivers, amongst other benefits (Bhatia et al., 2020). However, the studies reviewed did not include MS caregivers, and none were among the Irish population.

In the work of McKeown et al. (2004), MS caregivers within the Republic of Ireland and Northern Ireland expressed difficulties accessing information about relevant formal supports. Although peer support was not this study’s focus, the fact that MS caregivers identified other carers as one of their sources of information, with whom they engaged with at carer support meetings, is of interest (McKeown et al., 2004), highlighting the potential of peer support interventions in promoting information dissemination. However, as this study was conducted over two decades ago, there is a need to assess current experiences with peer support among this cohort.

To address this gap, this cross sectional mixed-methods study aimed to examine the needs and experiences of MS caregivers in Ireland relating to peer support, both in-person and online, factors influencing their engagement, and the associations between peer support, caregiver burden, loneliness, social support and wellbeing. With high MS prevalence of 193 per 100,000 persons in Ireland (Multiple Sclerosis International Federation, 2023), this study’s findings potentially can enhance the provision of supports for PwMS and their families. Findings may be particularly relevant to organisations such as MS Ireland (a national organisation involved in providing a range of supports for people affected by MS), and Family Carers Ireland (an organisation which has a broader remit supporting all family carers).

## Methodology

### Design and setting

The study involved an online cross-sectional mixed methods survey, collecting quantitative and qualitative data using a mixture of researcher-devised questions and validated measures. It had a convergent parallel design (Doyle et al., 2009), and collected complimentary information on peer support to gather a deeper understanding on the topic (Cresswell and Poth, 2016). Its reporting adhered to the Strengthening the Reporting of Observational Studies in Epidemiology (STROBE) cross-sectional reporting guidelines (von Elm et al., 2007).

The survey’s focus was deemed relevant and prioritised by a public and patient involvement (PPI) panel of three MS caregivers who differed in age, gender, caring relationships, and years spent caring for PwMS. Their participation followed an invitation to join a PPI panel, which was disseminated on the PPI Ignite Network and MS Ireland (MSI) websites. The former is collaboration between universities and organisations in Ireland promoting PPI innovation, while the latter is a national organisation providing a range of support services to PwMS and their family members living in Ireland. Interested MS caregivers responded to this invitation and met with the lead researcher (JA) to discuss the research project, selecting the focus from a range of potential options. They then contributed to reviewing and refining survey questions for clarity and relevance to MS caregivers in November 2023. PPI panel members received a small (€30) gratuity for their contributions in the form of a gift voucher. The participation of the PPI panel in the design and review of the study contributed to the survey’s relevance and minimised potential harm.

### Recruitment and data collection

The study received ethical approval from the Maynooth University Social Research Ethics Sub-Committee in January 2024 (Ethics Review ID: 37823). Participation was open to adults above the age of 18 years, living in the Republic of Ireland, who were informal MS caregivers. A caregiver was defined as anyone (spouse, partner, family member or friend) who provides any form of care, help or support to someone living with MS, to mitigate eligibility and inclusionary misunderstanding. Exclusion criteria included anyone not meeting the inclusionary criteria and formal caregivers who were paid to provide care.

Purposive and snowball sampling were used. A pragmatic sample size was anticipated, given the exploratory nature of this study and the hard-to-reach MS caregiver population (Lakens, 2022). Recruitment took place in February–March 2024 following the dissemination of a study advertisement through the social media platforms and mailing lists of MSI to increase participation of people who might have been missed. Additionally, participants could receive a forwarded advertisement from other participants known to them. The study advertisement gave a link to the survey, which was hosted on Qualtrics Software (Qualtrics, 2020) and preceded by an information sheet and online consent form. Participants providing full informed consent were made aware that their participation was anonymous, confidential, and non-completion was without penalty. Participants were also signposted to relevant resources to mitigate distress from participation. Data was collected as part of a larger study also involving PwMS who were presented with similar questions (Alaboson et al., 2025).

### Measures

#### Sociodemographic background

Participants were asked basic sociodemographic questions about their age, gender, residence, ethnicity, living situation, employment status, relationship status, and ease of making ends meet (measured on a 6-point scale where participants reported the extent to which they could make ends meet, ranging from ‘*with great difficulty’* (1) to ‘*very easily*’ (6)).

#### Caregiving characteristics

Participants specified their relationship to their care-recipient and quantified care-hours provided by answering the question, ‘*For how many hours per week do you provide care*?’. For guidance, participants were informed that 24-hour, 7 days a week care equated to a total of 168 hours per week.

#### Validated instruments

Table 1 summarises instruments that measured (1) caregiver burden, using the four-item version of the Zarit Burden Interview (ZBI: Bédard et al., 2001), (2) social support, using the three-item Oslo Social Support Scale (OSLO-3; Bøen et al., 2012), (3) loneliness, through the UCLA Loneliness Scale (version 3; Hughes et al., 2004), and (4) wellbeing, through the five-item World Health Organisation Wellbeing Index (WHO-5: Topp et al., 2015). Given the small sample size, Cronbach’s alphas were not calculated.

**Table 1.** Validated instruments.

Validated instrument	Psychometric properties	Instrument description	Total scores	Scoring categories
4-Item ZBI	Acceptable internal consistency ~ Cronbach’s α = 0.69–0.89 (Higginson et al., 2010)	Higher scores equivalent to higher burden, measured in Likert scale ‘Never’ (0) to ‘Nearly always’ (4)	0–16	0–7 ~ none to little
				8–12 ~ mild to moderate
				13–16 ~ severe
OSLO-3	Acceptable internal consistency ~ Cronbach’s α = 0.640 (Kocalevent et al., 2018)	It explores close contacts, perception of concern others show, and access to neighbourly help.	3–14	3–8 ~ poor
				9–11~ moderate
				12–14 ~ strong
UCLA-3	Unidimensional structure with good internal consistency and reliability ~ Cronbach’s α ranging from 0.89 to 0.94 (Russell, 1996)	It explores companionship, isolation, and feeling of exclusion in Likert scale responses ranging from ‘Hardly ever’ (1) to ‘Often’ (3).	3–9	3–4 ~ low
				5–6 ~ moderate
				7–9 ~ high
WHO-5	Unidimensional structure with good internal consistency ~ Cronbach’s α ranging from 0.81 to 0.85 (Fung et al., 2022)	Higher wellbeing corresponds to higher scores with enquiries about the preceding 2 weeks; Likert scale of 0 (‘At no time’) to 5 (‘All the time’)	0–100(*standardised by multiplying by 4*)	0–28 ~ risk of major depressive episodes
				29–50 ~ depression risk (Löwe et al., 2004; Topp et al., 2015)

#### Peer support

Participants were provided with a definition of peer support (i.e. ‘Peer support is an exchange of support between people who have a similar life experience (e.g. support from other caregivers of someone living with MS). Peer support can take place in person (e.g. meet-ups arranged by MS Ireland) or online (e.g. through social media, like MS Facebook groups, or through other online programmes)’). Following this definition, participants were asked a number of questions relating to their experiences of both in-person and online peer support. A range of researcher-devised open- and close-ended questions explored their experiences and expectations of in-person and online peer support. Participants were asked about the frequency of their engagement, their preferences for mode of engagement (in-person, teleconferencing platforms, websites, and other digital formats), examples of peer support they had received, perceived usefulness, benefits and disadvantages. They also provided information regarding their preferences for engaging in online group peer support including the composition of groups, ideal number of participants, and their preferences for anonymity.

### Data analysis

A mixed-methods approach was used to analyse survey data. Descriptive statistics were calculated for all variables, including frequencies and percentages of categorical variables, and means and standard deviations for continuous variables. Spearman rank correlation coefficients were also calculated to examine relationships between wellbeing, loneliness, social support, and frequencies of in-person and online peer support engagements, with significance levels set at 0.05. Missing data was excluded from the analysis.

Open-text responses explaining barriers to participation in peer support and reasons for non-engagement in both in-person and online peer support were coded with an inductive approach, and analysed through content analysis (Braun and Clarke, 2006; Erlingsson and Brysiewicz, 2017; Naeem et al., 2023). Data was coded using NVivo Version 14 (Smith, 2023) by JA and reviewed by RM/LC. Disagreements were discussed and arbitrated by discussion and consensus between JA, RM, and LC. Data triangulation was conducted at the interpretation stage (Doyle et al., 2009). Triangulation was chosen to maximise the strengths of the quantitative and qualitative aspects of the study, and provide a more nuanced picture by the qualitative data validating quantitative findings (Doyle et al., 2009).

## Results

### Descriptive statistics

Twenty-seven MS caregivers completed the survey. Only valid responses were included in the analyses.

#### Sociodemographic characteristics

Participants mainly identified as White or Caucasian (*n* = 24, 88.9%). On average, participants were 49.41 years of age (SD = 17.76), and predominantly female (*n* = 21, 78%), married (*n* = 17, 68%) and living in urban residences (*n* = 18, 72%). Other caregiver sociodemographic characteristics are summarised in Table 2.

**Table 2.** Participant sociodemographic and caregiving characteristics.

Variable	Category	Frequency (*n* = 27)	Valid percentage (%)	Missing, *n* (%)
Gender	Male	6	22	-
	Female	21	78	
Ethnicity	Caucasian	24	88.9	-
	Irish traveller	1	3.7	
	Mixed	1	3.7	
	Other (*Irish*)	1	3.7	
Residence	Rural	7	28	2 (7.4)
	Urban	18	72	
Employment status	Working full-time	8	33.3	3 (11.1)
	Working part-time	7	29.2	
	Homemaker	2	8.3	
	Retired	4	16.7	
	Student	3	12.5	
Relationship status	Married or cohabiting	17	68	2 (7.4)
	In a relationship but not cohabiting	2	8	
	Single	6	24	
Living situation	Living with family	25	100	2 (7.4)
Ease of making ends meet	Very easily	0		2 (7.4)
	Easily	9	36	
	Fairly easily	10	40	
	With some difficulty	5	20	
	With difficulty	1	4	
	With great difficulty	0		
Relationship to care recipient	Partner or spouse	17	70.8	3 (11.1)
	Parent	5	20.8	
	Sibling	2	8.3	
Age in years		49.41^a^ (17.76)^b^	[18,77]^c^	0 (0)
Care hours per week		82.17^a^ (72.3)^b^	[5168]^c^	3 (11.1)

a Mean.

b Standard deviation.

c Range.

#### Caregiving characteristics

Participants described providing care for an average of 82.17 hours per week (SD = 72.3), with a range of 5–168 hours weekly, highlighting high variability in care provision. The majority identified as a partner or spouse of their care recipient (*n* = 17, 70.8%), although other forms of relationships with care recipients were also reported (see Table 2).

#### Caregiver burden, social support, loneliness and wellbeing

Table 3 summarises caregiver experiences of burden, social support, loneliness and wellbeing along with their means and standard deviations (SD). Of note, there was an even split of caregivers reporting low and moderate caregiving burden, with none reporting high burden. Caregivers also reported low levels of loneliness, poor social support and lower than average wellbeing scores compared to the general population.

**Table 3.** Caregiver burden, social support, loneliness, and psychological wellbeing.

Variable/measure	Mean (SD)	Range	Category	*N*	Valid percentage (%)	Missing, *n* (%)
Caregiver burden/Zarit burden scale	7.08 (2.57)	1–11	Low	11	47.8	4 (14.8)
			Moderate	12	52.2	
Social support/OSLO 3	8.43 (2.25)	4–13	Poor	13	56.5	4 (14.8)
			Moderate	8	34.8	
			Strong	2	8.7	
Loneliness/UCLA-3	4.68 (1.96)	3–8	Low	14	63.6	5 (18.5)
			Moderate	1	4.54	
			High	7	31.8	
Wellbeing/WHO-5	47.09 (24.36)	16–92	Low or no depression risk	8	36.4	5 (18.5)
			Some depression risk	7	31.8	
			High depression risk	7	31.8	

SD: standard deviation; OSLO-3: the three-item Oslo social support scale; UCLA-3: The UCLA Loneliness Scale (version 3); WHO-5: World Health Organisation Wellbeing Index.

#### Peer support need and engagement

*Peer support need.* Just under a third of caregivers expressed no need for support from peers. The majority (63%) had an unmet need for support from peers (see Table 4).

**Table 4.** Caregiver peer support need and engagement.

Variable	Category	*N*	Valid percentage (%)	Missing, *n* (%)
Peer support need	I have no need for peer support	6	31.5	8 (29.6)
	I need peer support and these needs are currently met	1	5.3	
	I need peer support and these needs are currently unmet	12	63.2	
In-person peer support engagement	Never	17	77.3	5 (18.5)
	Rarely	4	18.2	
	Sometimes	1	4.5	
	Often	-		
	Always	-		
Online peer support engagement	Never	12	57.1	6 (22.2)
	Rarely	5	23.8	
	Sometimes	4	19	
	Often	-		
	Always	-		

#### Peer support engagement

As can be seen in Table 4, the majority of caregivers never engaged with other peers in person, while others did so sparingly, engaging either rarely or sometimes. Online engagement with peers was also limited. The majority of caregivers reported never engaging with other peers online. However, while online engagement was infrequent, it was slightly higher than in-person peer engagement.

### Associations between social support, loneliness, wellbeing and in-person and online peer support

A Spearman’s correlation matrix is presented in Table 5. Wellbeing had a significant medium positive relationship with social support (rho = 0.359, *p* = 0.047). There were no other significant associations observed. These included non-significant relationships: between (1) loneliness and peer support engagement, both in-person (rho = 0.357, *p* = 0.093) and online (rho = 0.409, *p* = 0.124), (2) wellbeing and peer support engagement, both in-person (rho = 0.199, *p* = 0.681) and online (rho = 0.206, *p* = 0.716), and (3) social support peer support engagement, both in-person (rho = 0.033, *p* = 0.831) and online (rho = −0.003, *p* = 0.124).

**Table 5.** Spearman’s rho correlations between social support, loneliness, wellbeing and in-person and online peer support (PS) engagement.

Variable	In-person PS engagement	Online PS engagement	UCLA-3	WHO-5	OSLO-3
In-person PS engagement	1.000				
Online PS engagement	0.095	1.000			
UCLA-3	0.357	0.409	1.000		
WHO-5	0.199	0.206	−0.056	1.000	
OSLO-3	0.033	−0.003	−0.316	0.359*	1.000

* *p* < 0.05.

### Open-text responses

Participants who had never participated in either online or in-person peer support were asked to provide the reasons preventing their engagement in open-text responses. Overall, 19 described in-person engagement barriers, and 11 described online barriers. Given the large overlap in responses, both in-person and online barriers were analysed together, with specific mention of additional online barriers.

### Barriers to peer support engagement

Two main themes: (1) limited awareness and availability of peer support, and (2) attitudes preventing peer support, were identified as the principal reasons for non-engagement with peers across both modalities. Table 6 summarises the open-text responses on barriers to peer support engagement.

**Table 6.** Reasons for non-engagement in peer support reported in open-text responses.

Themes	Sub-themes	*N*	Representative quotes
Both in-person and online barriers			
Limited awareness and availability of peer support (*n* = 15)	Unaware of supports	10	‘*I am not aware of other caregivers*’ ‘*Have not been told of caregiver groups*’
			‘*No communication from MS doctors and nurses, consultant and MS team*’
	Local support lacking	2	‘*Tried but nothing for us in the rural area where we live*’
	Little or no opportunities to connect with other caregivers	1	‘*Because I have very little contact with them*’
	No known (online) groups	2	‘*I don’t know of any support groups*’ ‘*Don’t see any*’
Attitudes preventing peer support (*n* = 10)	Uninterested in peer support	2	‘*Nor have sought them out*’ ‘*Never got involved and unsure why*’
	Peer support is not needed (in-person and online)	5	‘*I don’t feel the need*’
			‘*[I] just don’t*’
	Care recipients’ needs are prioritised instead	3	‘*I assume that their needs would be greater than* mine’
			‘*my husband is only 2* years *into his diagnosis, and we have struggled to find support for him and I - I feel that support for him is the priority*’
Specific online barriers			
Online engagement is challenging (*n* = 4)	Dislike digital engagement	2	‘*Don’t like doing things on social media*’ ‘*I don’t like it*’
	Lack digital participation skills	1	‘*Don’t know how to participate online*’
	Shy	1	‘*I’m shy and find it hard to get involved in things online*’
No time to engage in peer support (*n* = 2)	Busy	1	‘*Don’t have enough time*’
	Other responsibilities	1	‘*Busy with work and life*’

#### Theme 1: Limited awareness and availability of peer support

A total of 15 responses highlight this barrier to both in-person and online peer support engagement.

A notable number (*n* = 13) of responses highlighted an insufficient promotion of in-person peer support opportunities. The majority (*n* = 10) pointed to being unaware of available supports. Many expressed not knowing other MS caregivers or having opportunities to meet with them. Two responses highlighted no local opportunities to meet other MS caregivers. While one respondent noted that there is ‘*no support service in my area*’, another specified these disparities, describing their unsuccessful attempts to connect with peers around their rural residence: ‘*tried but nothing for us in the rural area where we live*’. In one instance, a caregiver stated that their non-engagement was ‘*because I have very little contact with them*’, suggesting that although they might know peers, support opportunities were not widely promoted or accessible. Then, highlighting another aspect of peer support promotion, one caregiver pointed out the need for signposting by healthcare workers, stating that there was ‘*no communication from MS doctors and nurses, consultant and MS team*’. Two responses expressed similar barriers to engagement with online peer support.

#### Theme 2: Attitudes preventing peer support

A total of 10 responses represented caregivers’ attitudes or beliefs that limited both in-person and online peer support engagement.

Six responses highlighted attitudes or beliefs that limited engagement with peers in-person. Despite its potential, not all respondents were interested in obtaining support from other caregivers (*n* = 2). They highlighted that they had ‘*never got involved and unsure why*’, and ‘*nor have sought them out*’. For some (*n* = 2), peer support was not needed. Their responses (‘*it has not arisen*’ and ‘*[I] just get on with myself*’) indicate that support from peers was not required, or that they felt adequate coping by themselves.

Finally, caregivers highlighted their care recipients’ needs were of higher priority, and the potential consequences of seeking support themselves. For example, one caregiver stated that ‘*my husband is only 2 years into his diagnosis, and we have struggled to find support for him and I - I feel that support for him is the priority*’, while another noted that ‘*my partner needs the support, and it might imply to my partner that he is a burden if I went to a support group for carers*’.

In addition, four responses revealed similar attitudes or beliefs that limited participation with peers online. One respondent described ‘*I don’t feel the need*’, while another was self-reliant, stating ‘*I prefer to deal with it personally*’. Another participant’s response highlighted they prioritised the care recipient’s need, saying ‘*I assume that their needs would be greater than mine*’. Finally, one response stated, ‘*[I] just don’t*’.

### Specific barriers to online engagement

Specific barriers to online peer support engagement were shared. Two themes, (1) online engagement is challenging, and (2) no time, were identified.

#### Theme 1: Online engagement is challenging

This theme acknowledges the peculiarities that online-mediated peer support might present to users, generally. The most common (*n* = 4) barrier to online engagement reported by caregivers was challenges navigating online platforms. Two respondents indicated a dislike for the online environment, for example stating that they ‘*don’t like doing things on social media*’. Other attitudes such as ‘*I’m shy and find it hard to get involved in things online*’ also limited engagement. Finally, one participant highlighted that they lacked the digital skills required to participate in online peer support stating, ‘[I] *don’t know how to participate online*’.

#### Theme 2: No time to engage in peer support

Two participants described either being busy or having other responsibilities, ‘*busy with work and life*’, that inadvertently prevented them from engaging with peers online.

### Data Triangulation

Both qualitative and quantitative findings indicated that caregivers need peer support, but do not know where or how to access it either in-person or online. Their experiences highlighted in open-text statements like ‘*I am trying to find support for me as a caregiver but have not found it so far*’ and ‘*I am not aware of other caregivers*’ can explain why a substantial number of caregivers (*n* = 12, 63.2%) indicated in their survey responses that these needs were unmet. On the other hand, some caregivers (*n* = 6, 31.5%) did not express a peer support need, with significant numbers in our sample reporting never having engaged in peer support either in-person (77.3%, *n* = 17) or online (57.1%, *n* = 12). Up to five respondents elaborated on this issue in their open-text responses: ‘*I don’t feel the need*’; ‘*I prefer to deal with it personally*’; ‘*[I] just don’t*’. These findings highlight how such attitudes may limit caregivers’ engagement in peer support, along with the prioritisation of their care recipients’ needs and a lack of interest in the support of peers.

Respondents provided fewer open-text responses regarding barriers to online engagement (i.e. six online compared to 25 in-person barriers), which included experiencing challenges navigating online environments or technology, or having no time. This is consistent with the finding that caregivers reported higher online than in-person engagement with peer support.

## Discussion

This study adds to our understanding of MS caregiver experiences and engagement with peer support, shedding light on deterrents to participation. Given the limited research on this topic to date, particularly in the Irish context, our findings have the potential to inform the development or adaptation of interventions to improve psychosocial outcomes among MS caregivers.

A key finding from our study was the fact that most caregivers expressed a need for peer support that often was not met. Peer support availability, either in locating other caregivers or peer support groups, was flagged as problematic. It might be that supportive programmes or events, such as those offered by MS Ireland, infrequently extend to family of PwMS or their social networks, thereby limiting opportunities for interaction among MS caregivers. However, even if such programmes were available, our findings of caregiver attitudinal hesitation suggest some caregivers may benefit from approaches that aim to support both PwMS and family members or partners that provide support. This could signal that caregiver needs also matter.

Existing research shows that generally MS caregivers have unmet needs (Borreani et al., 2014; Lorefice et al., 2013). Although there is limited research into the role peer support might play in meeting these needs, our findings suggest that this might be useful. Open-text responses in this study suggest that caregivers unsuccessfully seek this support, in line with their expectations of its utility. In other research, caregivers in neurodegenerative conditions report that peer support might alleviate stress and isolation, while providing targeted advice (Wallace et al., 2021), which might generally underlie why caregivers in our study express an unmet need for it. Caregivers may see peers as a safe place for each other, a community, where shared understanding of their circumstances prevails, and may then they go on to receive insights about their emotions and strategies towards better emotional regulation (Marziali and Donahue, 2006). These findings of social and emotional support, information and advice, are also mirrored in research focussing on a wider variety of conditions including cancer, mental health and addiction, where caregivers reported getting a sense of support to better provide care and feel support (Joo et al., 2022). Overall, these benefits may underpin the recognised peer support need among caregivers in this study.

Our study established that peer support needs prevail despite low experiences of loneliness, thus emphasising the unique support peers provide. Notably, the low levels of loneliness observed could be attributed to the relationships that participants had. For example, a notable number of participants identified as their care recipient’s partners and all lived with family, while over 60% were in some form of employment. However, while such relationships may provide social interaction, they are unlikely to address specific caregiver needs. When examining these findings through Mead et al.’s (2001) theoretical perspective on peer support, MS caregivers may recognise that contacts without MS caregiving experience cannot engage in meaningful listening, resulting in a mismatched perspective on MS-related topics. In addition, their contacts, though potentially alleviating feelings of loneliness, may not offer a safe space to reflect on their caregiving reality (Mead et al., 2001). In both instances, Dunn et al. (2003) highlight how emotional and appraisal supports are attributes that peers may provide.

Despite low experiences of loneliness, we found that caregivers also experienced low levels of social support and wellbeing compared to the general population. This contradiction could be investigated by confirming to what extent the instruments (OSLO-3 and UCLA-3) measured these constructs through a confirmatory factor analysis, although unfortunately the study sample size limits investigation. Beyond this, our findings might indicate that personal contacts play very distinct non-overlapping roles, providing companionship and presence compared to being available to help or showing concern. Research is needed to establish why caregivers may experience poor social support. This may include identifying the mismatch between caregivers’ social support expectations or desire, and their social network and family’s ability to meet them (McKeown et al., 2004), or the impact of caregiver co-morbidities, such as depression, on poor social support experiences (Bambara et al., 2014). Future research could also distinguish between social support and peer support barriers among MS caregivers. Such work would map MS caregivers’ perspective on the role of the wider social environment on their psychosocial wellbeing.

We found an expected, and significant positive relationship between social support and wellbeing, aligning with research showing that strong social support can protect caregivers from negative caregiving experiences and low psychological wellbeing (Cejalvo et al., 2021). Correspondingly, we found high levels of depressive risk in our participants who scored low on social support. This corroborates previous findings on the negative relationship between perceived MS caregiver social support and experiences of depression symptoms, regardless of the severity of MS symptoms in their care recipients (Bambara et al., 2014), or among care recipients with relapsing-remitting MS (Meca-Lallana et al., 2016), although limited by small sample sizes and their observational design. This study spotlights the potential value of interventions that improve social support and wellbeing, such as peer support (Lorefice et al., 2013).

We found low peer support engagement, where some caregivers in our study also expressed difficulties identifying other MS caregivers or available supports. There might be a tipping point where peer support need evolves to engagement. One of these might be MS severity of the care recipient. Although our study did not explore the role MS severity of care recipients play in peer support engagement, needs for information, psychosocial support, access to services have been demonstrated in other research where their care recipients lived with severe MS (Borreani et al., 2014). We know that these needs can be met by peer support, but little research has explored MS caregiver knowledge and or ability to navigate health and social systems to meet them. Further, there might be a distinct set of factors including cultural and social values that are associated with motivation to seek and or act on this need (Levesque et al., 2013), that have not been explored in this study but are echoed in the deterring attitudes expressed. More robust research is needed, since our findings are not unique to MS (Friedman et al., 2018).

We note that caregivers in our study reported experiencing low levels of wellbeing and peer support engagement, despite reporting only low to moderate caregiving burden. Participants reported variable but longer durations of care time, even up to 24 hours per day, yet perceived relatively low burden. While research shows that burden may contribute to both negative and positive quality of life in MS caregivers (Maguire and Maguire, 2020), our study established that most caregivers reported some-to-high depression risk, achieving low wellbeing scores overall. Although this may prompt peer support engagement, we found the opposite. Our study does not explore what demotivates peer support engagement. Future studies may delve into the role caregivers expect peers to play supporting their wellbeing and, given the wide range of activities that caregiving entails, explore why or what caregiving activities motivates engagement with peers.

Our findings emphasise barriers to peer support engagement. Participants expressed clearly that this support was unnecessary. The main barriers to engagement caregivers faced were difficulties locating or connecting to peer support, experiencing limiting attitudes, and not having the time or online skills to participate virtually. Other research has shown that caregivers also have low engagement with peers due to poor awareness or knowledge of opportunities to make contact with others, and fear of encountering others with more severe disability despite acknowledging its benefits and their need for such support (de Wit et al., 2019). Joo et al. (2022) noted that despite benefits, caregivers in their study could not avail of peer support because of time constraints and other responsibilities. While we have discussed the impact burden might have on peer support engagement, neither subjective nor objective burden was explored in their study, highlighting a potential avenue for future research. Similarly, a recent survey among informal caregivers of people living with dementia noted that participation in online peer support was related to their greater belief in its value (Yin et al., 2024). The dilemma some caregivers in our study faced in seeking peer support and prioritising their own needs over those of their care recipient, mirror other research where MS caregivers reported feeling guilty or even needing permission to engage in self-care (Wallace et al., 2021). Finally, the barriers to engagement in online peer support reported in the present study align with other research reporting that caregivers’ engagement can be limited by the ease of using online platforms and perceived quality of the support peers are able to provide (Wallace et al., 2021). Overall, the barriers identified in the present study appear to reflect similar sentiments with other caregiving literature, highlighting that these barriers might be applicable to caregivers more broadly.

Nonetheless, it is worth highlighting context-specific barriers in our research. For example, the fact that most MS caregivers were concerned with a lack of information about in-person opportunities to meet peers, including locally, with one caregiver pointing out their expectation to have received support information from their contact health workers, emphasises the need for more harmonised resources for people affected by MS in Ireland. In a separate study amongst PwMS and community workers in Ireland, PwMS reported a need for signposting to resources and services, and that in addition to meeting their needs, community workers also linked their caregivers with services or peers (Maguire et al., 2023), suggesting one way in which services can be extended to meet the needs of MS caregivers also. A study exploring non-use of cancer informational support services in Ireland also found that people desiring formal general supports expected to be signposted to them by their healthcare team (Sheridan et al., 2020). There is a potential for both health and social support systems to work more collaboratively in meeting needs in the community and increasing awareness of available supports.

Participants recounted the challenges they experienced in engaging with peer support online; either they disliked the experience or reported being shy or lacking digital skills. The use of technology and the internet may present unique frustrations that compound negative peer support experiences. This spotlights a potential issue amongst the wider MS caregiving population, especially those with limited digital access and skill, or low internet literacy. In a study evaluating an online peer support programme for caregivers in amyotrophic lateral sclerosis, participants cited challenges such as internet fluctuations and a lack of privacy that offset its potential to improve accessibility (Olesen et al., 2022). Additionally, online engagement requires a different set of skills that caregivers may not have or be able to acquire at the time peer support is critically needed, leading to disenfranchisement (Wallace et al., 2021). Our findings overall indicate the need for accessible MS information and collaborative resources targeting MS caregivers specifically as well as those they provide care for.

### Limitations

Participants were not asked about MS progression in their family or partner living with MS. MS progression in PwMS along with an increased need for assistance or support may impact on their caregiver wellbeing and or experiences of burden (Buchanan et al., 2010). This relationship can be elucidated in other studies. The cross-sectional design of this survey limits the exploration of causality. Studies with more methodologically robust designs can explore causality between burden, social support, loneliness, and wellbeing in caregivers. Further, limited qualitative data was collected. Future qualitative research should build upon this. For instance, in-depth interviews with caregivers may provide a better insight into the issues raised in this study.

Findings from this study are not generalisable and should be taken cautiously. First, the small sample size limits the ability to conduct relevant tests of associations and directionality of relationships. Consequently, only descriptive statistics and correlation analyses were deemed appropriate to summarise the findings. Second, given the nature of the recruitment and data collection procedure this online study might have favoured caregivers who had greater engagement with digital and or social media content. Thus, a greater reflection of those that do not or have limited digital engagement could have been omitted.

Finally, there was significant homogeneity in participants’ demographic characteristics. For example, most of the sample identified as female Caucasians, who were in a relationship, and lived in urban residences across Ireland. While taking on the caregiving role tends to disproportionately affect females globally (World Health Organisation, 2024), and within Ireland (Central Statistics Office, 2022), it is expected that males would play a significant role in caregiving for PwMS given the predominance of this condition in women (Multiple Sclerosis International Federation, 2020). As such, the large proportion of females in our sample is at odds with that found in other research in this cohort. For example, a cross-sectional survey of over 1,300 MS caregivers in North America found participation of predominantly male caregivers (McKenzie et al., 2015). Contrary to expectations, participants were also predominantly female spouses of PwMS. It is possible a greater proportion of male spouses or caregivers might have shed light on gender differences reflected in previous literature suggesting that men and women have differing caregiving experiences (McKenzie et al., 2015).

### Implications

As few studies have explored the experiences of MS caregivers in relation to peer support, this study provides valuable insights into the needs and experiences of this cohort, as well as their perceptions of social support, loneliness, caregiving burden, and wellbeing. While findings highlight an often unmet need for peer support, this need is not universal. Future research should expand on our findings and explore, quantitatively and qualitatively, what motivates this need and how it might change over time, for example with MS progression. There is also a need for research to explore whether peer support interventions can improve caregiver wellbeing, and how this might impact PwMS and their quality of life and relationships.

For organisations such as MSI, and the wider cohort of MS healthcare practitioners, these findings highlight a need to recognise the role that caregivers and families play in the lives of PwMS, and their potential need for signposting to organisations that provide support to caregivers. Given this study’s findings, we advocate for the development of peer support interventions that address MS caregivers’ needs, in line with the recognition of the impactful role of informal caregivers and their contribution to healthcare costs in the context of MS (World Health Organisation, 2024).

## Conclusion

This mixed methods study explored the experiences of caregiving burden, social support, loneliness, wellbeing and peer support among MS caregivers in Ireland. The findings highlighted low participation in peer support among this cohort, despite a demonstrated need for it. MS caregivers cite poor promotion of available peer supports and low value placed on peer support as general barriers to engagement. In the case of engaging in online opportunities, MS caregivers may be limited by a lack of appropriate digital skills. Overall, there is a need for future research to explore these findings more robustly, along with the development of interventions that focus on MS caregivers to improve their wellbeing.
